# Lung Cancer: Understanding Its Molecular Pathology and the 2015 WHO Classification

**DOI:** 10.3389/fonc.2017.00193

**Published:** 2017-08-28

**Authors:** Kentaro Inamura

**Affiliations:** ^1^Division of Pathology, The Cancer Institute, Japanese Foundation for Cancer Research, Tokyo, Japan

**Keywords:** adenocarcinoma, driver mutation, genetic alteration, histology, molecular pathology, lung cancer

## Abstract

Lung cancer is the leading cause of cancer-related death worldwide due to late diagnoses and limited treatment interventions. Recently, comprehensive molecular profiles of lung cancer have been identified. These novel characteristics have enhanced the understanding of the molecular pathology of lung cancer. The identification of driver genetic alterations and potential molecular targets has resulted in molecular-targeted therapies for an increasing number of lung cancer patients. Thus, the histopathological classification of lung cancer was modified in accordance with the increased understanding of molecular profiles. This review focuses on recent developments in the molecular profiling of lung cancer and provides perspectives on updated diagnostic concepts in the new 2015 WHO classification. The WHO classification will require additional revisions to allow for reliable, clinically meaningful tumor diagnoses as we gain a better understanding of the molecular characteristics of lung cancer.

## Introduction

Lung cancer is the leading cause of cancer-related deaths worldwide in both men and women (1). It is categorized into two main histological groups: small cell lung carcinoma (SCLC, 15% of all lung cancers) and non-SCLC (NSCLC, 85% of all lung cancers). NSCLCs are generally subcategorized into adenocarcinoma, squamous cell carcinoma (SqCC), and large cell carcinoma. Accumulating evidence suggests that lung cancer represents a group of histologically and molecularly heterogeneous diseases even within the same histological subtype (2–26).

The histopathological classification of lung cancer has recently been revised and published as the 2015 WHO classification (2). Several major revisions were made in this classification to reflect recent discoveries related to the molecular pathology of lung cancer.

Human comprehensive molecular characterization projects have resulted in the identification of novel molecular characteristics of lung cancer and the different subtypes at levels of DNA alteration, DNA methylation, mRNA expression, microRNA expression, and protein expression. This review introduces and briefly summarizes recent studies on the molecular pathology of lung cancer with a focus on the association between molecular profiles and morphology (2).

## The 2015 Who Classification

The WHO classification was updated based on newly identified molecular profiles and targetable genetic alterations in lung cancer. For lung adenocarcinoma, the 2011 International Association for the Study of Lung Cancer, American Thoracic Society, and European Respiratory Society classification (27) was mostly adopted in the 2015 WHO classification. Table 1 shows the WHO classification of lung tumors (epithelial tumors) (2). The major revisions to the WHO classification are described below.

**Table 1:** WHO classification of tumors of the lung (epithelial tumors) (2).

Adenocarcinoma	Large cell carcinoma
Lepidic adenocarcinoma	Adenosquamous carcinoma
Acinar adenocarcinoma	Pleomorphic carcinoma
Papillary adenocarcinoma	Spindle cell carcinoma
Micropapillary adenocarcinoma	Giant cell carcinoma
Solid adenocarcinoma	Carcinosarcoma
Variants of adenocarcinoma	Pulmonary blastoma
Invasive mucinous adenocarcinoma	Other and unclassified carcinomas
Mixed invasive mucinous and non-mucinous adenocarcinoma	Lymphoepithelioma-like carcinoma
Colloid adenocarcinoma	NUT carcinoma
Fetal adenocarcinoma	Salivary gland-type tumors
Enteric adenocarcinoma	Mucoepidermoid carcinoma
Minimally invasive adenocarcinoma	Adenoid cystic carcinoma
Non-mucinous	Epithelial–myoepithelial carcinoma
Mucinous	Pleomorphic adenoma
Preinvasive lesions	Papillomas
Atypical adenomatous hyperplasia	Squamous cell papilloma
Adenocarcinoma *in situ*	Exophytic
Non-mucinous	Inverted
Mucinous	Glandular papilloma
Squamous cell carcinoma (SqCC)	Mixed squamous cell and glandular papilloma
Keratinizing SqCC	Adenomas
Non-keratinizing SqCC	Sclerosing pneumocytoma
Basaloid SqCC	Alveolar adenoma
Preinvasive lesion	Papillary adenoma
SqCC *in situ*	Mucinous cystadenoma
Neuroendocrine tumors	Mucous gland adenoma
Small cell carcinoma	
Combined small cell carcinoma	
Large cell neuroendocrine carcinoma (LCNEC)	
Combined LCNEC	
Carcinoid tumors	
Typical carcinoid	
Atypical carcinoid	
Preinvasive lesion	
Diffuse idiopathic pulmonary neuroendocrine cell	
Hyperplasia	

### Definition of Adenocarcinoma and SqCC

**Figure 1:**
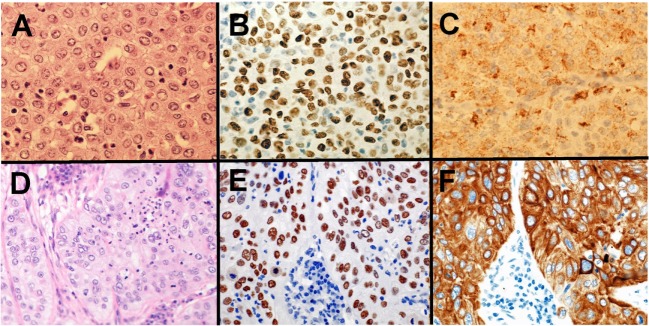
Solid adenocarcinoma **(A–C)** and non-keratinizing squamous cell carcinoma (SqCC) **(D–F)**. Solid adenocarcinoma [**(A)** HE staining] is immunohistochemically positive for TTF-1 **(B)** and Napsin A **(C)**. Non-keratinizing SqCC [**(D)** HE staining] is immunohistochemically positive for p40 **(E)** and CK5/6 **(F)**.

Pathologists are required to categorize lung cancer into adenocarcinoma and SqCC due to the targetable driver genetic alterations identified in lung adenocarcinoma and inappropriate drugs for SqCCs due to side effects in patients with SqCC. Before the 2015 WHO classification, adenocarcinoma was defined as carcinoma with an acinar/tubular structure or mucin production, whereas SqCC was defined as carcinoma with keratinization or intercellular bridges. If poorly differentiated carcinoma lacking light microscopic evidence of glandular differentiation (Figure 1A) is proven by immunohistochemistry to express “adenocarcinoma markers,” such as TTF-1 (Figure 1B) and/or Napsin A (Figure 1C), it is diagnosed as a solid adenocarcinoma. If poorly differentiated carcinoma lacking light microscopic evidence of squamous differentiation (Figure 1D) is proven by immunohistochemistry to express “SqCC markers,” (28) such as p40 (Figure 1E), CK5/6 (Figure 1F), and p63, it is diagnosed as non-keratinizing SqCC. Because of this classification, the proportion of large cell carcinoma has been markedly reduced.

### Adenocarcinoma

#### Classification according to the Extent of Invasiveness

The 2015 WHO classification divides adenocarcinomas into adenocarcinoma *in situ* (AIS, preinvasive lesion), minimally invasive adenocarcinoma (MIA), or (overt) invasive adenocarcinoma based on the extent of invasiveness. The disease-free survival rate of AIS and MIA when completely resected is 100% (29).

Adenocarcinoma *in situ* is defined as an adenocarcinoma comprising a lepidic pattern with a diameter of ≤3 cm. If the tumor diameter exceeds 3 cm, it is defined as “lepidic predominant adenocarcinoma, suspect AIS” because these tumors are rare and lack adequate characterization.

Minimally invasive adenocarcinoma is defined as an adenocarcinoma with a diameter of ≤3 cm and an invasion size of ≤5 mm. Even if the tumor size and invasion size comply with the definition of MIA, the presence of lymphovascular invasion, pleural invasion, or tumor necrosis can be an exclusion factor for an MIA diagnosis. If the tumor size exceeds 3 cm with an invasion size of ≤5 mm, it is defined as “lepidic predominant adenocarcinoma, suspect MIA” because these tumors are rare and lack adequate characterization.

The term “invasive adenocarcinoma, mixed subtype” for invasive adenocarcinoma is no longer used. Invasive adenocarcinoma is now classified using five predominant patterns: lepidic, papillary, acinar, micropapillary, and solid adenocarcinoma.

#### Variants of Invasive Adenocarcinoma

The term “mucinous bronchioloalveolar carcinoma (BAC)” is no longer used because most mucinous BACs included invasive components. Therefore, the term “invasive mucinous adenocarcinoma (IMA)” replaced mucinous BAC. IMA and mucinous AIS are accurately classified based on invasiveness. Besides IMA, variants of invasive adenocarcinoma comprise enteric, colloid, and fetal adenocarcinoma. Enteric adenocarcinoma is defined as adenocarcinoma with a predominant component that resembles adenocarcinoma arising in the colorectum and often shows CDX2 immunoreactivity (30).

### Squamous Cell Carcinoma

In the 2015 WHO classification, SqCCs are classified into keratinizing SqCC, non-keratinizing SqCC, and basaloid SqCC. Before this classification, basaloid SqCC was categorized as a variant of large cell carcinoma. However, basaloid SqCC immunohistochemically shows “SqCC markers” (e.g., p40, CK5/6, and p63) and is therefore categorized as SqCC.

### Neuroendocrine Tumors

In the 2015 WHO classification, a new category of “neuroendocrine tumors” was established. Invasive neuroendocrine tumors comprise three subtypes: SCLC, large cell neuroendocrine carcinoma (LCNEC), and carcinoid tumor (typical/atypical). Diffuse idiopathic pulmonary neuroendocrine cell hyperplasia is extremely rare and non-invasive; therefore, its clinical importance is low. On the other hand, the distinction between a high-grade neuroendocrine tumor (HGNET), comprising SCLC and LCNEC, and a carcinoid tumor is very important in both pathological and clinical practice. HGNET is one of the most aggressive subtypes and characterized by a history of heavy smoking in the patient, whereas carcinoid tumors usually carry a benign prognosis and frequently occur in patients with no history of smoking.

## Comprehensive Molecular Profiling

With the emergence of high-throughput sequencing techniques, detailed molecular profiles of lung cancer have been identified. The Cancer Genome Atlas (TCGA) research network identified genomic and other molecular alterations among a number of different types of cancer, including lung cancer. In this section, the comprehensive molecular profiles of lung cancer, mainly determined by TCGA, are introduced.

### Adenocarcinoma

The comprehensive molecular profiling of 230 lung adenocarcinoma by TCGA was published in 2014 (3). The authors reported high rates of somatic mutations (mean: 8.9 mutations per megabase) and identified 18 statistically significant genetic mutations: *TP53* (46%), *KRAS* (33%), *KEAP1* (17%), *STK11* (17%), *EGFR* (14%), *NF1* (11%), *BRAF* (10%), *SETD2* (9%), *RBM10* (8%), *MGA* (8%), *MET* (7%), *ARID1A* (7%), *PIK3CA* (7%), *SMARCA4* (6%), *RB1* (4%), *CDKN2A* (4%), *U2AF1* (3%), and *RIT1* (2%).

Furthermore, approximately 75% of the lung adenocarcinomas examined harbored genetic alterations that promote the RTK/RAS/RAF signaling pathway. Of all the cases, 62% showed driver genetic alterations that promote the RTK/RAS/RAF pathway. Among them, mutations in *KRAS, EGFR*, and *BRAF* comprised 32, 11, and 7.0%, respectively. Other genetic alterations that promote the RTK/RAS/RAF pathway included *MET* exon 14 skipping (4.3%), *ERBB2* (or *HER2*) mutation (1.7%), *ROS1* fusion (1.7%), *ALK* fusion (1.3%), *MAP2K1* mutation (0.9%), *RET* fusion (0.9%), *NRAS* mutation (0.4%), and *HRAS* mutation (0.4%). An examination of the DNA copy number of the remaining 38% cases without driver genetic alterations that promote the RTK/RAS/RAF pathway revealed amplification of oncogenes in the RTK/RAS/RAF pathway: *ERBB2* amplification (0.9%) and *MET* amplification (2.2%). The authors also identified new genetic alterations in this pathway: mutations in *NF1* and *RIT1*. *NF1* is a tumor suppressor gene that regulates the RTK/RAS/RAF pathway, and the frequency of *NF1* mutations was 8.3%. Similarly, *RIT1* constitutes a part of the RTK/RAS/RAF pathway, and the frequency of *RIT1* mutations was 2.2%. Consequently, 75% of lung adenocarcinomas have genetic alterations that promote the RTK/RAS/RAF pathway. This study on the comprehensive molecular characterization of lung adenocarcinoma widened the potential therapeutic targets of lung adenocarcinoma.

This study conducted mRNA profiling and provided new transcriptional subtypes: i.e., the terminal respiratory unit (TRU, formerly bronchioid), the proximal-inflammatory (PI, formerly squamoid), and the proximal-proliferative (PP, formerly magnoid) mRNA subtypes. The TRU subtype was enriched for *EGFR* mutation and kinase fusions. The PI subtype was characterized by solid morphology and co-mutation of *NF1* and *TP53*. The PP subtype was enriched for *KRAS* mutation and *STK11* inactivation.

DNA methylation profiling divided lung adenocarcinomas into three subtypes: i.e., CpG island methylator phenotype (CIMP)-high, CIMP-intermediate, and CIMP-low subtypes. CIMP-high tumors often showed DNA hypermethylation of *CDKN2A, GATA2, GATA5, HIC1, HOXA9, HOXD13, RASSF1, SFRP1, SOX17*, and *WIF1*. The CIMP-high subtype was enriched for *MYC* overexpression as well as for DNA hypermethylation of genes in WNT pathway.

Protein profiling divided lung adenocarcinomas into six subtypes. The top 50 differentially expressed proteins among the 6 subtypes included Cyclin D1, Smad4, p-mTOR, Rad50, beta-Catenin, and HER2. The six subtypes partially overlapped with the mRNA three subtypes.

### Squamous Cell Carcinoma

The comprehensive molecular profiling of 178 cases of SqCC by TCGA was published in 2012 (4). As can be expected from the history of heavy smoking in SqCC patients, SqCCs are characterized by complex genomic alterations. The authors indeed detected a mean of 360 exonic mutations, 165 genomic rearrangements, and 323 segments of copy number alteration per tumor. They identified 11 statistically significant genetic mutations: *TP53, CDKN2A, PTEN, PIK3CA, KEAP1, MLL2, HLA-A, NFE2L2, NOTCH1, RB1*, and *PDYN*. The frequency of *TP53* mutations was 90%. The authors identified novel loss-of-function mutations in the *HLA-A* class I major histocompatibility gene. They also conducted pathway analyses and identified pathways related to oxidative damage, including *KEAP1* and *NFE2L2* in 34% of cases, squamous cell differentiation pathway, including overexpression of *SOX2* and *TP63* in 44% of cases, PI3K/AKT pathway in 47% of cases, and inactivation of *CDKN2A* in 72% of SqCC cases. These results provided us multiple potential targets for the treatment of lung SqCC.

The mRNA profiling divided SqCCs into four subtypes: i.e., classical, basal, secretory, and primitive subtypes. The classical subtype was characterized by pronounced hypermethylation, chromosomal instability, and alterations in *KEAP1, NFE2L2*, and *PTEN*. The basal subtype showed *NF1* alterations. The primitive subtype was enriched for *RB1* and *PTEN* alterations.

MicroRNA profiling divided SqCCs into four subtypes. These four subtypes roughly overlapped with the mRNA four subtypes. DNA methylation profiling also divided SqCCs into four subtypes (methylation clusters 1–4). Methylation cluster 4 showed little DNA hypermethylation and included most of the primitive mRNA subtype. Cluster 3 was predominantly made up of the classical mRNA subtype and enriched for *NFE2L2* mutations. Cluster 3 and Cluster 2 showed the highest levels of DNA hypermethylation. Cluster 1 showed intermediate DNA hypermethylation levels.

### Small Cell Lung Carcinoma

In 2012, comprehensive genomic analyses were reported by two groups. Rudin et al. identified *SOX2* as a frequently amplified gene in SCLC (6). *In vitro* suppression of *SOX2* blocked the proliferation of *SOX2*-amplified SCLC cell lines. Furthermore, they identified a recurrent *RLF–MYCL1* fusion in SCLC with RNA sequencing. *In vitro* silencing of *MYCL1* in SCLC cell lines with an *RLF–MYCL1* fusion decreased cell proliferation. On the other hand, Peifer et al. reported recurrent mutations in genes that encode histone modifiers, including *CREBBP, EP300*, and *MLL*, suggesting histone modification as a major feature of SCLC (7).

In 2015, George et al. sequenced the genome of 110 SCLCs and found comprehensive genomic profiles (5). SCLC is characterized by highly complex genomic alterations, and C:G>A:T transversions were found in 28% of all mutations on average, which is a characteristic pattern of heavy smoking. Almost all examined SCLCs showed bi-allelic inactivation of *TP53* and *RB1*. Genomic alterations of tumor suppressor gene *TP73* was observed in 13% of SCLCs. Among *TP73* genomic alterations, genomic rearrangement of *TP73Δex2/3* was identified. Because *TP73Δex2/3* promotes carcinogenesis, *TP73Δex2/3*-targeted strategy is a promising treatment for SCLC. The authors also observed inactivating mutations of *NOTCH* family genes (31), which suppressed neuroendocrine differentiation *via* the regulation of *ASCL1* expression in 25% of SCLCs.

The mRNA profiling divided SCLCs into two groups (5). Most of SCLCs (83%) were categorized into group 2, which was characterized by higher expressions of *CHGA, GRP, ASCL1*, and *DLK1*. Group 1, comprising 17% of SCLCs, showed lower expressions of these four genes.

## Histology and Genetic Profiles

Close associations exist between histology/morphology and genetic profiles. In this section, several of these associations are introduced.

### Driver Genetic Alterations and Histology of Lung Adenocarcinoma

*EGFR* mutation is one of the most common driver mutations in lung adenocarcinoma, and *EGFR*-mutated adenocarcinoma is characterized by East-Asian ethnicity, female gender, and non/light-smoking history (32). Pathologically, *EGFR*-mutated lung adenocarcinomas typically show nuclear TTF-1 (NKX2-1) immunostaining and a hobnail cell type. Adenocarcinomas with a micropapillary pattern have a higher frequency of *EGFR* mutations than adenocarcinomas without this pattern (33, 34).

Fusion genes were recently identified as oncogenic drivers. In lung adenocarcinoma, rearranged genes, including *ALK, ROS1, RET, NTRK1*, and *NRG1*, have been reported. *ALK*-rearranged adenocarcinoma comprises 4–5% of adenocarcinomas (35). *ROS1*- and *RET*-rearranged adenocarcinoma each comprises approximately 1% (35, 36). These rearranged adenocarcinomas show a good clinical response to molecular-targeted drugs. *ALK*-rearranged adenocarcinoma is characterized by a TTF-1 cell lineage, an acinar structure with mucin/signet-ring cell pattern, non-/light-smoking history, and young onset (37–39). *ROS1*- and *RET*-rearranged adenocarcinomas have a similar histology to *ALK*-rearranged adenocarcinoma, such as mucinous cribriform pattern or solid signet-ring cell pattern (35, 40, 41).

*NTRK1* fusion in lung adenocarcinoma was identified by Vaishnavi et al. (42) A *NTRK1*-rearranged adenocarcinoma identified by Shim et al. belonged to IMA subtype (43). NTRK1 is a TRKA kinase; thus, TRKA kinase inhibitors have the potential to be used in the treatments of *NTRK1*-rearranged adenocarcinomas. *NRG1*-rearranged adenocarcinoma is also characterized by IMA (44, 45). IMAs are frequently *KRAS*-mutated; therefore, Nakaoku et al. examined 34 IMA cases without *KRAS* mutations, partly by whole-transcriptome sequencing (45). They identified five oncogenic fusions: *CD74–NRG1, SLC3A2–NRG1, EZR–ERBB4, TRIM24–BRAF*, and *KIAA1468–RET*. These fusion genes were mutually exclusive from *KRAS* mutations. *NRG1* fusions were present in 17.6% (6/34) of *KRAS*-wild-type IMAs. Because fusions of *NRG1, ERBB4, BRAF*, and *RET* are potential molecular targets, their clinical applications are promising.

Driver genetic alterations in lung adenocarcinomas differ between Caucasians and Asians, and between smokers and non-smokers. For example, *KRAS* mutations are frequently detected in lung adenocarcinomas in smokers, whereas genetic alterations in *EGFR, ALK, ROS1*, and *RET* are frequently detected in lung adenocarcinomas in non-smokers. The frequencies of driver genetic alterations in Caucasians were determined in TCGA study (3), whereas lung adenocarcinoma in Asians is characterized by a high frequency of *EGFR* mutations (approximately 50%) and low frequency of *KRAS* mutations (approximately 10%).

### MicroRNAs and Histological Subtypes of Lung Adenocarcinoma

MicroRNAs are small single-stranded non-coding RNAs (19–22 nucleotides in length) that play important regulatory roles, including lung carcinogenesis (46–50). Nadal et al. performed microRNA profiling of adenocarcinomas subclassified by the 2015 WHO classification (51). They demonstrated that different histological subtypes of lung adenocarcinoma have distinct microRNA expression profiles. Unsupervised hierarchical clustering divided adenocarcinomas into three major clusters, which correlated with the histological subtypes of the 2015 WHO classification. Cluster 1 included fewer acinar and solid adenocarcinomas, and nearly all the tumors in cluster 1 were categorized as lepidic adenocarcinomas or IMAs. In contrast, clusters 2 and 3 included more acinar and solid adenocarcinomas and fewer lepidic adenocarcinomas and IMAs. Solid adenocarcinoma was characterized by the overexpression of *miR-27a, miR-212*, and *miR-132* (51).

Enteric adenocarcinoma is one of the new variants of lung adenocarcinoma in the 2015 WHO classification. It is defined as an adenocarcinoma with a predominant component that shows enteric differentiation (30). Garajová et al. found that the microRNA signature of enteric adenocarcinomas shows similarities with NSCLCs and pancreatic adenocarcinomas, but not with colorectal adenocarcinomas. Enteric adenocarcinomas share oncogenic microRNAs (miR-31*, miR-126*, miR-506, miR-508-3p, and *miR-514*) with pancreatic adenocarcinomas (52).

### MicroRNAs and SCLC

Small cell lung carcinoma, which is categorized into neuroendocrine tumor, shows high expression of ASCL1, which is a transcription factor that promotes neuroendocrine differentiation. A study reported that *miR-375* expression was promoted by ASCL1 in lung neuroendocrine carcinoma (53). This study suggested that *miR-375* might reduce the YAP1-associated proliferative arrest by inhibiting YAP1. Another study examined microRNAs from 50 SCLC patients and 30 healthy individuals, and suggested that level of *miR-92a-2* in plasma could be a potential non-invasive method for the SCLC diagnosis (54). Recent evidence suggests that *miR-21* expression may be higher in HGNET (i.e., SCLC and LCNEC) than in typical/atypical carcinoid, and that high expression of *miR-34a* may be associated with atypical carcinoids (55).

## Molecular Alterations and Their Therapeutic Relevance

Molecular alterations, which have been recently elucidated in lung cancer, are used in clinical practice or potentially useful therapeutic tools in the treatment of lung cancer.

In lung adenocarcinoma, fusions of *ALK, RET*, and *ROS1* have been shown to be targetable genetic alterations (35, 36). In IMA, new fusions of *NTRK1, NRG1, ERBB4, BRAF*, and *RET* were identified as targetable genetic alterations (43, 45). The comprehensive molecular profiling in lung adenocarcinoma by TCGA research network has shown that 75% of lung adenocarcinomas have genetic alterations that promote RTK/RAS/RAF pathway; these genetic alterations included newly identified *NF1* and *RIT1* mutations, both of which are potentially targetable (3).

For lung SqCC, no effective targetable agents have been developed specifically. The comprehensive molecular profiling in lung SqCC by TCGA research network has identified a potential therapeutic target in the most lung SqCCs that they investigated (4). The alterations in targetable oncogenic pathways in lung SqCCs included RTK pathway (26%), RAS pathway (24%), and PI(3)K pathway (47%) (4). Targeting these pathways are potential ways of the treatment for lung SqCC.

Small cell lung carcinoma is the deadliest subtype of lung cancer, and no established molecular-targeted therapy for SCLC exists. For SCLC, recent studies discovered potentially targetable genetic alterations, including *SOX2* amplification, *RLF-MYCL1* fusion, and *TP73Δex2/3* (5, 6).

In the era that we have comprehensive genomic data of lung cancer, we should try to treat lung cancer thorough more efficacious targeted therapeutic interventions.

## Conclusion and Future Directions

This review focuses on newly identified molecular pathology and the 2015 WHO classification of lung cancer, which was revised based on the better understanding of the molecular pathology of lung cancer and recent advancements in newly developed molecular-targeted drugs. However, in an era of precision medicine, these classification changes remain inadequate. In the near feature, the WHO classification will need to be further revised to allow for reliable, clinically meaningful tumor diagnoses that reflect our better understanding of the molecular characteristics of lung cancer.

## Author Contributions

KI conceived the review, wrote the text, created the table and figure, and approved the final draft.

## Conflict of Interest Statement

The author declares that the research was conducted in the absence of any commercial or financial relationships that could be construed as a potential conflict of interest.
